# Multidisciplinary study of thorium mobility: formation of turkestanite and steacyite analogues, and structural insights using an XRD-directed microcrystal preparation technique

**DOI:** 10.1107/S2052520625004822

**Published:** 2025-07-04

**Authors:** M. Stachowicz, B. Bagiński, D. E. Harlov, P. Jokubauskas, J. Kotowski, W. Matyszczak, A. Dąbrowska, R. Macdonald

**Affiliations:** a Department of Geochemistry, Mineralogy and Petrology, Faculty of Geology University of Warsaw 02-089Warsaw Poland; b GFZ Helmholtz Centre for Geosciences, Telegrafenberg, 14473Potsdam, Germany; c Faculty of Earth Resources China University of Geosciences Wuhan430074 People’s Republic of China; d Department of Geology University of Johannesburg PO Box 524 Auckland Park2006 South Africa; e Spectroscopy of Nanomaterials, Faculty of Chemistry University of Warsaw Pasteura 1 Warsaw02-093 Poland; f Biological and Chemical Research Centre University of Warsaw Żwirki i Wigury 101 st. Warsaw02-089 Poland; g Environment Centre Lancaster University Lancaster LancashireLA1 4YQ United Kingdom; Academy of Sciences of the Czech Republic, Czechia

**Keywords:** microcrystal preparation, single-crystal X-ray diffraction, turkestanite, steacyite, hydro­thermal alteration, rare earth element

## Abstract

Minerals of the ekanite group typically contain Th, U or REE (rare earth elements) as primary structural constituents. The *^C^*Na analogues of turkestanite, *^A^*Th*^B^*(Ca,Na)*^C^*(K_1–*x*_□_*x*_)*^T^*(Si_8_O_20_) (□ is vacancy), and steacyite, *^A^*Th*^B^*(Ca,Na)*^C^*(K_1–*x*_□_*x*_)*^T^*(Si_8_O_20_), isostructural minerals of the ekanite group, were experimentally synthesized during hydro­thermal alteration of chevkinite-(Ce). The experiment was conducted at 550°C, 200 MPa, at an oxygen fugacity approximately equivalent to the Ni–NiO (NNO) buffer with NaF and Ca(PO_4_)_2_ added to the hydrous fluid. Both phases formed as a replacement of chevkinite-(Ce) and earlier alteration products. Their identity was confirmed by electron probe microanalysis and electron backscatter diffraction as *^C^*Na analogues of turkestanite and steacyite. Further SCXRD, and Raman spectroscopy analyses confirmed that it is a new *^C^*Na analogue with respect to the ekanite mineral group. Raman spectroscopy revealed the presence of H_2_O within the crystal structure. A dedicated FIB workflow was designed to extract single crystals ready for SCXRD analysis. Compositionally, the phase is Na rich and depleted in K, REE and Fe. The mean formula based on 20 O atoms can be written as *^A^*(Th_0.94_U_0.03_)_0.97_*^B^*(Na_0.96_Ca_0.90_Mn_0.11_Ce_0.02_Nd_0.01_Fe_0.01_)_2.0_*^C^*(Na_0.83_K_0.07_)_0.9_*^T^*Si_8.05_O_20_·0.1*^C^*(H_2_O). It crystallizes in space group *P*4/*mcc* with *a* = 7.4757 (2) Å, *c* = 14.9658 (7) Å, *V* = 836.38 (6) Å^3^, and *Z* = 2. Compositional variation is represented mainly by the relationship Ca^2+^ + □ → 2Na^+^, where □ is a vacancy which can also be filled by H_2_O during crystallization. The synthesis from this study represents the first record of *^C^*Na analogues of turkestanite and steacyite. A dedicated microcrystal selection technique is presented allowing for easy single-crystal X-ray diffraction.

## Abstract 2

A novel focused ion beam and scanning electron microscope-based technique has been developed for mounting microcrystals on carbon fibre, making them immediately compatible with single-crystal X-ray diffraction. This method has been successfully applied to studying the crystal chemistry of a synthetic Th-bearing, Na analogues of turkestanite and steacyite, and should prove suitable for the study of other micro-sized phases.

## 1. Introduction

Investigations of the structure of natural Th-bearing silicates are quite limited as these silicates are often in a poor crystalline state due to metamictization. Due to this poor crystal quality and the limited number of natural examples, the crystal chemistry of Th silicates has not received much attention. Given the geological significance of Th related to REE ores, its potential as a nuclear fuel source (Allibert *et al.*, 2015 ▸) and its role in waste immobilization (Ewing, 1976 ▸), understanding the inorganic crystal chemistry of Th-bearing phases is valuable (Mann *et al.*, 2015 ▸). The Th-bearing silicates turkestanite (Tkt), *^A^*Th*^B^*(Ca,Na)*^C^*(K_1–*x*_□_*x*_)*^T^*(Si_8_O_20_), and steacyite (Scy), *^A^*Th*^B^*(Na,Ca)*^C^*(K_1–*x*_□_*x*_)*^T^*(Si_8_O_20_), where □ = vacancy, are members of the ekanite group of minerals (Hawthorne *et al.*, 2019 ▸). There is a disagreement in the literature regarding the presence and location of H_2_O in the mineral’s structure. Only Pautov *et al.* (1997 ▸) have reported the presence of H_2_O. Other structural studies have not detected H_2_O in turkestanite (Kaneva *et al.*, 2023 ▸; Kabalov *et al.*, 1998 ▸; Petersen *et al.*, 1999 ▸) or steacyite (Richard & Perrault, 1972 ▸). In steacyite, Na is the dominant cation in the *B* site, whereas in turkestanite, *^B^*Ca is dominant.

Turkestanite is known from a small number of localities, including the Dzhelisu Massif (Kyrgystan) (Pautov *et al.*, 1997 ▸; Kabalov *et al.*, 1998 ▸), Dara-i-Pioz (Tajikistan) (Pautov *et al.*, 1997 ▸; Kaneva *et al.*, 2023 ▸), the Ilímaussaq complex (Greenland) (Petersen *et al.*, 1999 ▸), the Papanduva pluton in the Morro Redondo complex (Brazil) (Vilalva & Vlach, 2010 ▸), and the Ambohimirahavavy complex (Madagascar) (Estrade *et al.*, 2018 ▸). The main occurrences are in alkaline complexes, such as peralkaline granites. Steacyite has been identified in Mont Saint-Hilaire (Richard & Perrault, 1972 ▸; McDonald & Chao, 2004 ▸; Grice & Gault, 2006 ▸; Lykova *et al.*, 2024 ▸) and Rouma Island in the Los Archipelago (Guinea) (Biagioni *et al.*, 2010 ▸). Turkestanite in the Papanduva pluton in the Morro Redondo complex formed under subsolidus conditions buffered to magnetite–hematite at temperatures of 450°C, when earlier crystallization of Na and HFSE-rich (high-field strength elements such as Zr, Hf, Nb, Th, U, REE) minerals led to the enrichment of the residual melts in Ca (Vilalva & Vlach, 2010 ▸). At Dara-i-Pioz, Kaneva *et al.* (2023 ▸) described a pegmatoidal rock composed dominantly of quartz, albite and aegirine, with turkestanite as an accessory phase. In a GR-III granite from the Ambohimirahavavy complex, Tkt takes the form of an early magmatic phase, occurring as euhedral crystals enclosing albite, K-feldspar, Na-clinopyroxene and Na-amphibole (Estrade *et al.*, 2018 ▸).

Tkt or Scy analogues have been previously synthesized in a series of experiments at 450 or 500°C, 450 to 610 MPa over 16 days, which were aimed at determining the stability relationships between monazite, fluorapatite, allanite and REE-epidote, utilizing Na_2_Si_2_O_5_ + H_2_O as the reactive fluid (Budzyń *et al.*, 2011 ▸).

In this study, a series of high *P*–*T* experiments involving hydro­thermal alteration of the REE-Ti silicate chevkinite-(Ce), synthetic *^C^*Na analogues of turkestanite (Na-Tkt) and steacyite (Na-Scy) formed in one experiment (numbered CF-15) was undertaken. This provided us with the opportunity to (i) add to the geochemical database on the mineral; (ii) provide structural data on it; (iii) add some constraints on the *P*–*T* conditions under which it forms; (iv) show where H_2_O is likely to substitute; (v) show whether Na can occupy the *C* site in a 12-coordinated cage; and (vi) determine whether REE are more likely to substitute for Ca on the *B* site rather than Th on the *A* site.

From a purely analytical perspective, we describe a straightforward procedure to extract µm-sized crystals for single-crystal X-ray diffraction (SCXRD) using a scanning electron microscope (SEM) coupled and a Ga focused ion beam (FIB). Beyond the ability to apply SCXRD to materials for which larger crystals are unavailable, the essentially extinction-free data from microcrystals enable high-precision determination of electron densities and vibrational amplitudes (Rieck *et al.*, 1988 ▸). The mounting of microcrystals is becoming increasingly important as advances in X-ray free-electron lasers, microfocus synchrotron beamlines and even laboratory diffractometers now enable data collection from crystals with dimensions of only a few µm or less (Wagner *et al.*, 2013 ▸; Takaba *et al.*, 2024 ▸; Guo *et al.*, 2018 ▸). Observing such small crystals effectively under a stereo-microscope is challenging since the defects are not as visible compared with imaging in a SEM using secondary electron mode. In this study, we propose a procedure that eliminates the need for an optical stereo-microscope. Crystals selected using a SEM can be directly mounted onto a goniometer head, streamlining the process and reducing the risk of damaging or losing delicate microcrystals during transfer. By utilizing FIB and nano­manipulation techniques, researchers can precisely extract and prepare microcrystals, thereby expanding the scope of materials that can be studied and enhancing the accuracy of structural determinations.

## 2. Experimental alteration of chevkinite-(Ce)

The starting material for the experiment was pristine chevkinite-(Ce) from the Diamer district in Pakistan. The composition of the Chev-(Ce) starting material is given in the supporting information The formula can be written as: (Ce_1.85_La_0.79_Nd_0.64_Ca_0.39_Pr_0.22_)_3.9_Fe^2+^(Fe^2+^_1.03_Ti_0.75_Mn_0.16_)_1.9_Ti_2_(Si_2_O_7_)_2_O_8_. This is close to the standard formula for chevkinite: *A*_4_Fe^2+^(Fe^2+^Fe^3+^Ti)_2_Ti_2_(Si_2_O_7_)_2_O_8_ (Ito & Arem, 1971 ▸). Thorium oxide contents ranged between 0.54 and 2.89 wt%, averaging 0.94 wt%.

The experiment (CF-15) was undertaken at the Deutsches GeoForschungsZentrum in Potsdam. The pressure and temperature used, 200 MPa and 550°C, reflect the *P–T* conditions under which chevkinite generally experiences alteration, *i.e.* in the upper crust. The experimental duration was 84 days. The experimental charge consisted of H_2_O (5 mg), NaF (2 mg), Ca_3_(PO_4_)_2_ (5 mg), natural Amelia albite (Ab_99_; 5 mg), quartz (5 mg), and chevkinite (15 mg), which was added to a 1.5 cm long, 3 mm wide Pt capsule. This feldspar–quartz assemblage broadly simulates the mineralogy commonly found in the host rocks of chevkinite (syenites and granites).

The experiment was carried out using a standard cold seal autoclave in conjunction with a hydro­thermal high-pressure line. An internal thermocouple was placed such that its tip was located half-way up along a sealed platinum capsule placed at the end of the autoclave. The thermocouple was accurate to within ±3°C with a maximum thermal gradient of ∼5°C along the 1.5 cm length of the capsule. Pressure on the hydro­thermal line was calibrated against a pressure transducer calibrated against a Heise gauge manometer. After the run, the autoclave was quenched using compressed air. A temperature of 100°C was reached within 1 min. The contents of the platinum capsule were lightly fragmented and embedded in ep­oxy. The resulting grain mount was then evaluated using scanning electron microscopy, electron probe microanalysis (EPMA), and electron backscatter diffraction (EBSD) at the Laboratory of Electron Microscopy, Microanalysis and X-Ray Diffraction, Faculty of Geology, University of Warsaw.

## 3. Analytical methods

### 3.1. Scanning electron microscopy and EPMA

Detailed imaging at high magnifications and preliminary phase identifications were performed using a ZEISS AURIGA 60 FE (field emission) SEM equipped with two Bruker Xflash 6|30 energy-dispersive silicon drift detectors. Electron probe microanalysis based on wavelength dispersive spectrometry (WDS) was conducted on selected crystals with a Cameca SXFiveFE electron probe microanalyzer. The sample was cleaned and carbon-coated (∼20 nm) prior to analysis using a single carbon thread on a Leica EM ACE200 carbon coater in pulse mode. Results from EPMA for Na-Tkt and Na-Scy are given in Table 1 ▸. All details concerning analytical conditions are available in the supporting information (raw analyses section).

### 3.2. EBSD

The grain mount was additionally polished using a vibrating polisher for eight hours in a diamond suspension with a grain diameter of 0.25 µm. The sample was cleaned and carbon-coated (∼4 nm) prior to analysis using a single carbon thread on a Leica EM ACE200 carbon coater in pulse mode. EBSD patterns were collected with a Zeiss Auriga electron microscope equipped with a Bruker e-FlashHR+ detector with an integrated ARGUS imaging device. The sample was tilted to 70° using a dedicated stage (tilt about sample *X*-axis) for an optimal EBSD signal with a working distance set to 22.7 mm. The detector tilt angle was 2.42° and the sample-to-detector distance was 16.14 mm. EBSD was preceded by system calibration, where the sample to detector distance and pattern centre position were determined. EBSD was carried out using an electron beam with an accelerating voltage of 20 kV. Image tilt correction was performed with Zeiss *SmartSEM *software and no image rotation was applied. Each EBSD pattern had 400 × 300 pixel resolution. The system was calibrated in *ESPRIT 2.1* (Bruker). The collected patterns were compared to theoretical and simulated Kikuchi lines generated by *ESPRIT 2.2* (Bruker) software. A representative experimental pattern for Na-Tkt and the corresponding simulated pattern (right) are shown in Fig. S1.

### 3.3. Raman spectroscopy

**Figure 1:**
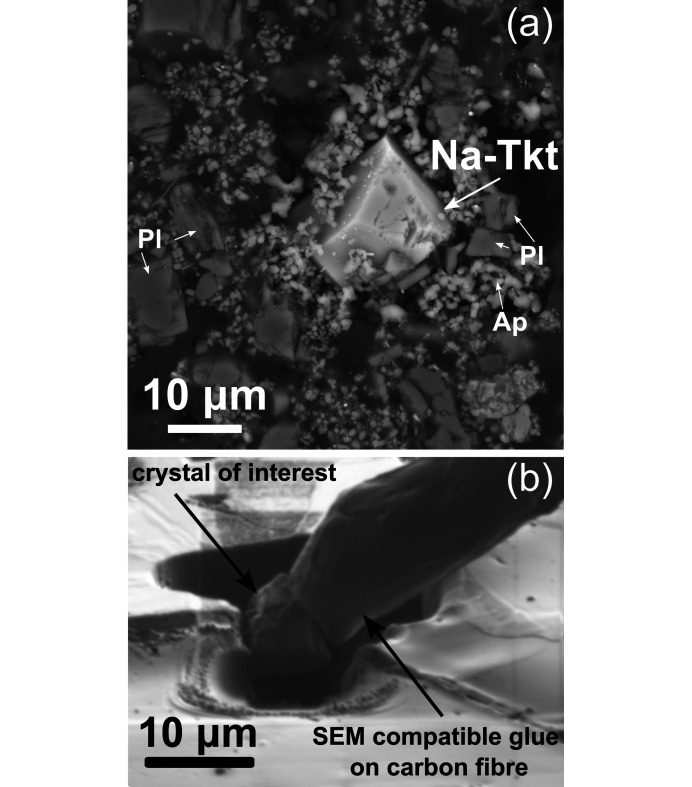
Euhedral *^C^*Na analogue of turkestanite (Na-Tkt) in matrix including apatite (Ap) and alkali feldspar (Pl) (*a*). It was later extracted using FIB for SCXRD analysis (*a*). The final stage of the crystal separation procedure (*b*). The area around and below the crystal was cut using FIB. The crystal was attached to the carbon fibre with SEM-compatible glue. The crystal holder was specifically designed to be compatible with the subsequent SCXRD experiment.

Analyses were carried out using a Renishaw inVia Qontor Raman spectrometer with 785 nm laser excitation, 50 mW laser power, 1800 lines per mm grating and a Peltier-cooled CCD detector. Single spot analyses were made using a 100× objective lens. The analysis was conducted on a crystal embedded in a polished ep­oxy mount, as shown in Fig. 1 ▸(*a*). The crystal was later extracted in the SEM chamber for SCXRD.

Since the investigated phase contains REE, laser-induced photoluminescence artefacts could interfere with Raman measurements, potentially leading to misinterpretation of spectral features (Lenz *et al.*, 2015 ▸). To distinguish between Raman bands and possible photoluminescence artefacts, we performed measurements using excitation laser wavelengths of 785 and 532 nm. However, using the 532 nm wavelength resulted in strong self-fluorescence, which obscured Raman features. Nevertheless, a weak but distinct Raman band at ∼3300 cm^−1^ was detected, indicating the presence of H_2_O. Following the approach suggested by Lenz *et al.* (2015 ▸), we performed additional measurements using a 785 nm laser, which minimized fluorescence and provided a clearer Raman spectrum. The final spectra presented here were obtained using the 785 nm laser.

### 3.4. Microcrystal extraction using SEM-FIB

FIB technology can assist in single-crystal preparation procedures; particularly in the extraction of microcrystals for SCXRD analysis [Fig. 1 ▸(*b*)]. With the introduction of advanced diffractometers and the increased intensity of X-ray radiation sources, along with continually improving detector sensitivity, it has become feasible to measure ever-smaller crystals in laboratory settings. This advancement is pivotal for crystallographic studies as it enables the detailed analysis of samples that were previously too small to be examined effectively or required synchrotron radiation. Additionally, the use of nanomanipulators within the SEM chambers, even without FIB, can be an invaluable tool in selecting single crystals with an ideal morphology from crystallized aggregates. Nanomanipulators allow for the isolation of single crystals, free from imperfections, ensuring that only the most suitable crystals are selected for analysis.

A small fragment (5 × 10 × 10 µm) of a Na-Tkt grain was extracted from experiment CF-15 [Fig. 1 ▸(*a*)] at the Laboratory of Electron Microscopy, Microanalysis and X-Ray Diffraction, Faculty of Geology, University of Warsaw using a Auriga Zeiss SEM equipped with a Ga^+^ ion gun, which was the source of a focused Ga ion beam, and a Kleindiek Nanotechnik nanomanipulator for an *in situ* lift-out. The crystal shown in Fig. 1a appears slightly larger than the final FIB-extracted fragment used for SCXRD, as the size changed during sample preparation. An ion beam with an accelerating voltage of 30 kV and current in the range of 28 nA to 1 nA were applied.

The extracted crystal was transferred *via* a nanomanipulator to a carbon fibre, itself attached to a metal pin from the manipulator probe [Fig. 1 ▸(*b*)]. A SEM compatible glue (Kleindiek Nanotechnik) was used to attach the carbon fibre to the crystal. The metal pin was then manually separated from the manipulator probe and placed on the goniometer base of the diffractometer. Crystal holder preparation details: a carbon fibre of ∼5 mm length was attached to a metal pin so that ∼2 mm of the carbon fibre extended beyond the end of the pin, serving as a holder for the crystal of interest. The carbon fibre was attached to this metal pin using cyano­acrylate glue. The metal pin was then mounted on the arm of a nanomanipulator. During the FIB procedure, the crystal was attached to the fibre using SEM-compatible glue from Kleindiek [Fig. 1 ▸(*b*)]. This glue remains flexible under low electron beam currents and hardens at higher magnifications and higher currents. After FIB-cutting, the metal pin, with the crystal of interest attached, can simply be removed from the nanomanipulator arm and transferred directly to the diffractometer.

### 3.5. SCXRD

Neither the SEM-compatible glue nor the carbon fibre produces visible background interference on the detector during the collection of diffraction frames. Moreover, the extraction of crystals did not affect the presence and position of H_2_O in the structure of the crystal. SCXRD was carried out using a SuperNova, four-circle diffractometer with Mo *K*α radiation (λ = 0.7148 Å) working at 50.00 kV and 0.80 mA, equipped with a CCD Eos detector (Rigaku Oxford Diffraction). The detector-to-crystal distance was 63.0 mm. A frame-width of 1° in ω scans and a frame time of 800 s (400 + 400) were used for data collection. Reflection intensities were corrected for Lorentz, polarization and absorption effects, and converted to structure factors using *CrysAlisPro* (v1.171.43.90; Rigaku Oxford Diffraction, 2023 ▸) software.

The observed unit-cell parameters (Table 1 ▸) for Na-Tkt are consistent with tetragonal symmetry. The statistical analysis of the |*E*| distribution yields |*E*^2^ – 1| = 0.931, indicating a centrosymmetric structure. The suggested space group is *P*4/*mcc*, supported by the presence of systematic absences consistent with glide planes. Scattering curves for neutral atoms are taken from *International Tables for Crystallography* (Prince, 2004 ▸). The following curves were used: Th at the *A* site; Ca versus Na at the *B* site; Na versus K at the *C* site; Si at the *T* site; and O at the O1–O3 sites. The *T* and O sites were found to be fully occupied by Si and O, respectively. The *B* and *C* sites have a mixed occupancy (0.55 Ca, 0.45 Na) and (0.96 Na, 0.04 K), respectively. The refined occupancy of the *A* site is 98% of Th. Details of the data collection and refinement are given in Table 2 ▸. Assigned site populations are collected in Table 3 ▸. Bond valences, calculated based on the bond valence parameters of Gagné and Hawthorne (2015 ▸), are shown in Table 4 ▸. Additional experimental details and the structural model are supplied in the crystallographic information file (cif) and also in Tables S3 and S4 (supporting information).

## 4. Results and discussion

### 4.1. Formation of synthetic *^C^*Na analogues of turkestanite and steacyite

**Figure 2:**
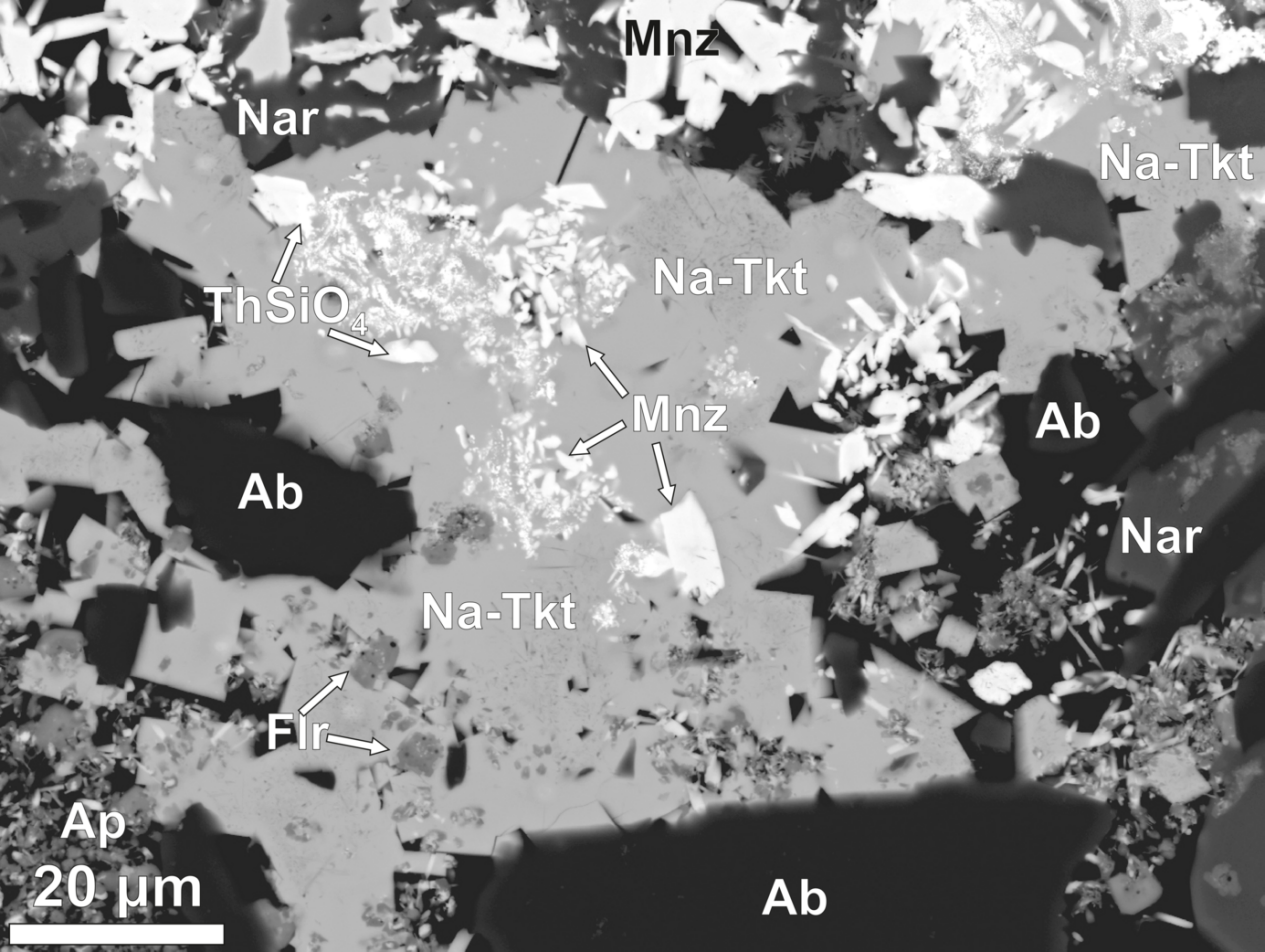
Backscatttered electron (BSE) image of large (∼80 µm across) crystal of *^C^*Na analogue of turkestanite (Na-Tkt), with inclusions of monazite (Mnz), fluorite (Flr), albite (Ab) and a ThSiO_4_ phase. It is uncertain whether monazite predated, or is intergrown with, Na-Tkt. Experiment CF15.

**Figure 3:**
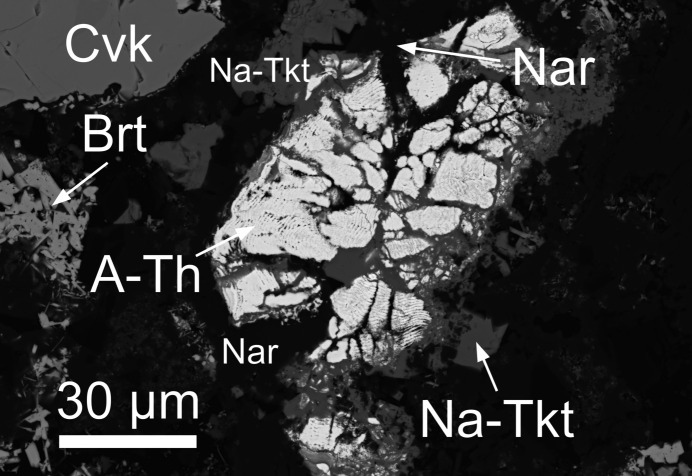
BSE image of the *^C^*Na analogue of turkestanite (Na-Tkt) rimming the amorphous ThSiO_4_ phase (A-Th). Brt – britholite-(Ce); Nar – narsarsukite; Cvk – chevkinite-(Ce)

Since the phases formed during experimental alteration are synthetic products, they should not be considered minerals without clarification. However, for simplicity, these phases will be referred to using mineralogical terminology. Although both *^C^*Na analogues of Tkt and Scy were identified in experiment CF-15, their compositions are nearly identical, differing only in the minor dominance of Na or Ca at the *B* site. For clarity and consistency, we refer to both as Na-Tkt throughout the text and figures. The earliest identified phase to form in experiment CF-15, taking the form of rims around the chevkinite-(Ce), was fluorbritholite-(Ce), which itself is partially rimmed by titanite. The enclosing matrix consisted of monazite-(Ce), Na-Tkt, narsarsukite [Na_2_(TiFe^3+^)Si_4_(O,F)_11_], fluorite and albite. Euhedral crystals are rare [Fig. 1 ▸(*a*)]. Normally, Na-Tkt occurs as anhedral crystals with inclusions of monazite, fluorite, albite and ThSiO_4_ (Fig. 2 ▸). It is uncertain whether monazite predated Na-Tkt, or was intergrown with it. In Fig. 3 ▸, the relationship between Na-Tkt and ThSiO_4_ is illustrated, where chevkinite-(Ce) has been completely replaced by amorphous ThSiO_4_, forming a laminar texture within the crystals. Subsequently, Na-Tkt replaced ThSiO_4_ along the rims and as veins, indicating a later formation stage. This spatial and temporal sequence suggests that the thorium required for Na-Tkt crystallization was sourced from the breakdown of ThSiO_4_ during its alteration.

Detailed studies on the formation and relationships between ThSiO_4_ phases, including huttonite, thorite and the amorphous form, from the same CF-15 and other experiments are presented in a separate study (Stachowicz *et al.*, 2024 ▸). Thorium exhibited considerable mobility under hydro­thermal conditions, particularly in alkaline fluids, where it likely formed stable complexes with hydroxyl and fluoride ions (Choppin & Jensen, 2006 ▸). The transformation from huttonite to thorite occurred at constant pressure and temperature, indicating kinetic control consistent with Ostwald’s step rule (Van Santen, 1984 ▸). At later stages, an amorphous ThSiO_4_ phase developed, most likely through fluid-driven hydration of thorite or huttonite, rather than through radiation-induced metamictization. The crystallization sequence observed in the experiment—huttonite, thorite, and finally the amorphous phase—suggested a progression controlled by both fluid composition and reaction kinetics. In addition, the microstructural arrangement of the precursor chevkinite-(Ce) appeared to influence the nucleation and orientation of the secondary thorite.

**Figure 4:**
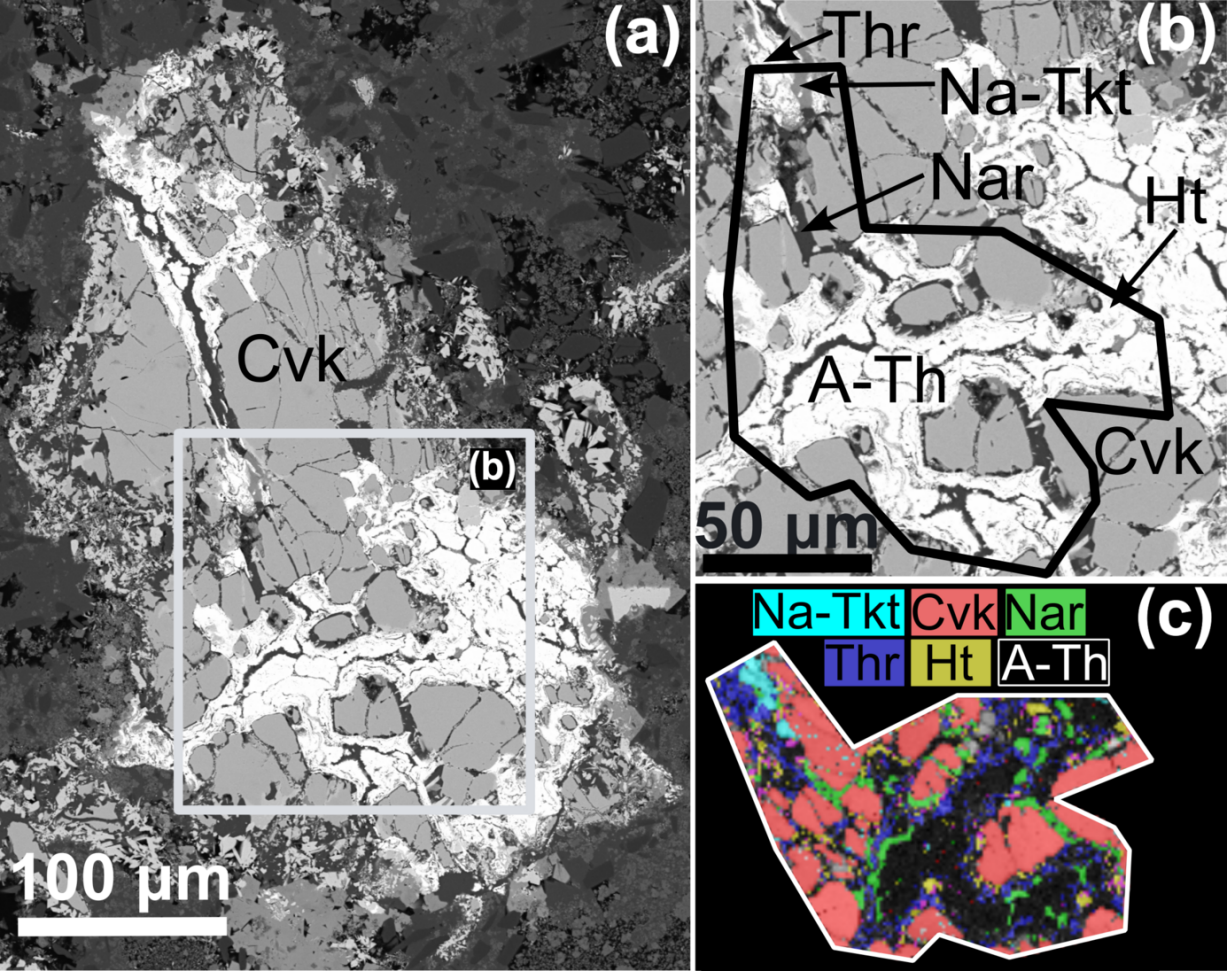
BSE image of chevkinite-(Ce) (Cvk) partially replaced by amorphous ThSiO_4_ (A-Th), huttonite (Ht) and thorite (Thr) and *^C^*Na analogue of turkestanite (Na-Tkt) in experiment CF15 (*a*). Thick black line indicates the area of EBSD analysis (*b*). Phase maps retrieved from EBSD measurement (*c*).

In experiment CF-15, Na-Tkt formed prismatic to anhedral grains intergrown with monazite, fluorite or feldspars. The phase identification was performed using EBSD, as shown in Fig. 4 ▸, which presents a BSE image of one of the areas analysed of the CF-15 reaction products, an image of the analysed regions and the resulting phase map.

### 4.2. Chemical composition of synthetic *^C^*Na analogues of turkestanite and steacyite

**Figure 5:**
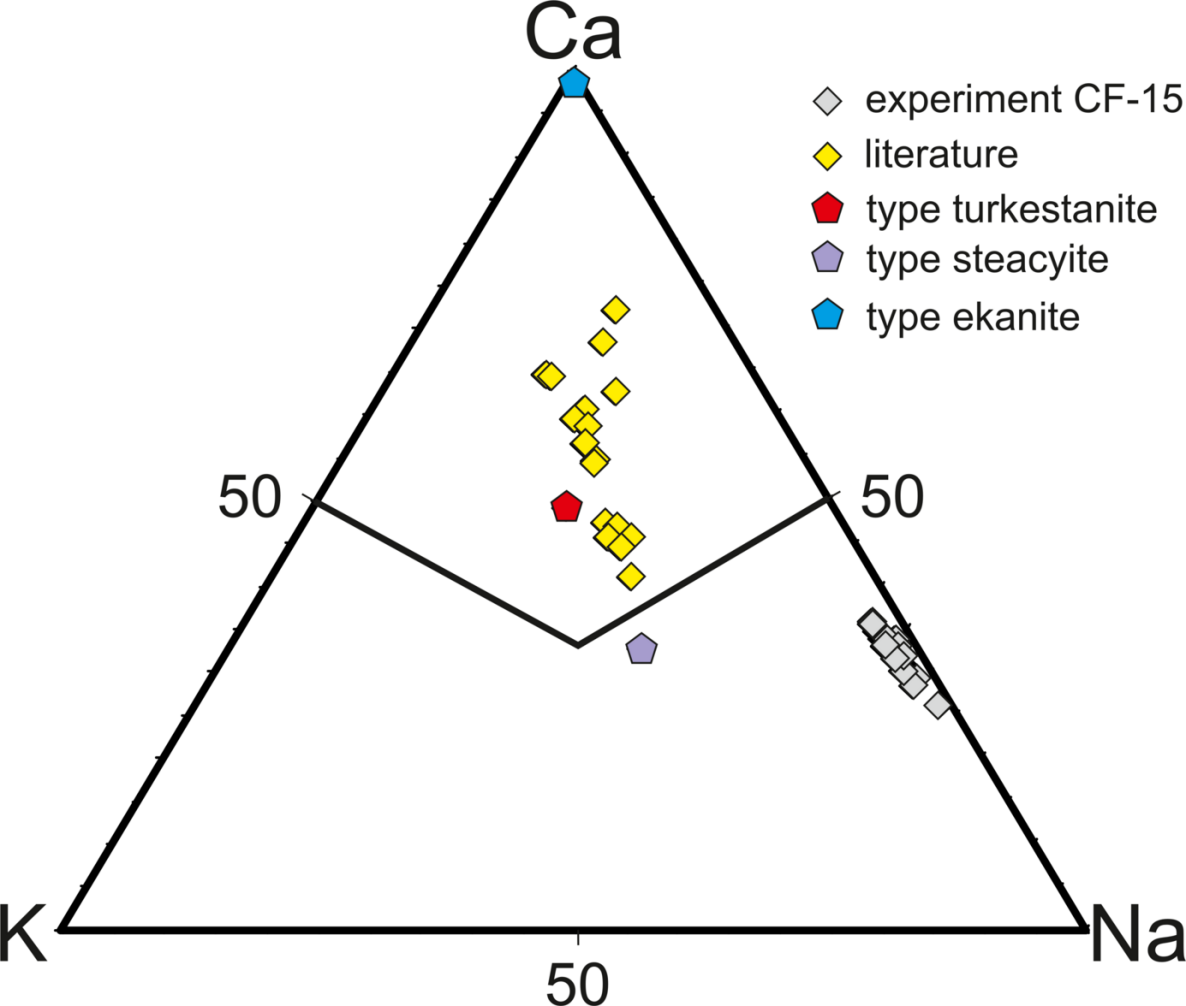
Compositional relationships of the Ca-Na analogue of turkestanite (grey diamonds, this study) compared with type turkestanite (red hexagons; Pautov *et al.*, 1997 ▸), steacyite (grey hexagons; Perrault & Szymański, 1982 ▸), and ekanite (blue hexagons; Anderson *et al.*, 1961 ▸) from the literature. Additional comparative data (yellow diamonds) include samples from the Morro Redondo Complex and Graciosa Province, Brazil (Vilalva & Vlach, 2010 ▸), Ambohimirahavavy complex, Madagascar (Estrade *et al.*, 2018 ▸), Dara-i-Pioz (Kaneva *et al.*, 2023 ▸), Jelisyiski Massif, and other Central Asian localities (Vilalva & Vlach, 2010 ▸), as well as experimental results (Budzyń *et al.*, 2011 ▸).

Results from EPMA for Na-Tkt are given in Table 2 ▸. Analytical totals are in the range 99.9–101.4 wt% (average 100.0 wt%). Formulae calculated on the basis of 20 O atoms give cation sums of 11.87 to 12.15 atoms per formula unit (apfu) (average 12.0 apfu), close to the stoichiometric value of 12. After filling the *B* site, excess Na is expected to enter the *C* site. The remaining deficit in the site (0.02–0.19) is taken to be occupied by H_2_O based on SCXRD and Raman spectroscopy (Section 4.3). The average formula can be written: *^A^*(Th_0.94_U_0.03_)_0.97_*^B^*(Na_0.96_Ca_0.90_Mn_0.11_Ce_0.02_Nd_0.01_Fe_0.01_)_2.0_*^C^*(Na_0.83_K_0.07_)_0.9_*^T^*Si_8.05_O_20_·0.1*^C^*(H_2_O). Vilalva & Vlach (2010 ▸) suggested that a possible coupled substitution scheme for Tkt is 2 Na^+^ + K^+^ + REE^3+^ ↔ 3 Ca^2+^ + □. The major coupled substitution scheme is *^B^*Na^+^ + *^C^*Na^+^ ↔ *^B^*Ca^2+^ + *^C^*□, which allows for the substitution of Na^+^ on the *B* site of up to 1 apfu. A possible minor coupled substitution scheme of REE is *^B^*Na^+^ + *^B^*REE^3+^ ↔ 2*^B^*Ca^2+^. These compositions are compared to the published analyses on a Ca–Na–K plot in Fig. 5 ▸, along with specimens of type Scy, ekanite and Tkt. Published analyses of these minerals form a broad trend from ekanite through Tkt towards Scy, suggesting complete solid solution between ekanite and Scy. Na-Tkt resulting from CF-15 is quite distinct from other Tkt or Scy analyses in that it is K-poor.

EBSD data for this phase show the best fit to the crystal structure of Tkt (Fig. S1), which supports *^C^*Na analogues of Tkt. This was due to the fact that the chevkinite-(Ce) was K-poor and the fluid was Na-rich. In addition, Vilalva & Vlach (2010 ▸) noted that Th(REE,Na)□(Si_8_O_20_) fitted the general formula of Scy and ekanite, which in essence predicts the occurrence of a K-free phase in the steacyite–ekanite–turkestanite mineral group. The subsequent SCXRD analysis partially supports these hypotheses, showing that the crystal structure of the phase studied here is almost free of K and isostructural. However, no full vacancy is present, and Na occupies this site instead.

### 4.3. Allocation of H_2_O and Raman spectroscopy

Raman spectroscopy is a well established analytical technique in geosciences (King & Mernagh, 2025 ▸), particularly valuable for identifying of REE-bearing minerals based on their characteristic vibrational spectra (Lenz *et al.*, 2015 ▸). In addition, diagnostic OH-related bands above 3100 cm^−1^ can provide critical insights into structural dynamics, as their intensities and shifts in Raman position are sensitive to temperature-induced changes within the crystal structure (Środek & Dulski, 2025 ▸). Among these, the band near 3590 cm^−1^ is typically attributed to strongly bonded OH groups and exhibits limited temperature sensitivity. In contrast, the band at approximately 3390 cm^−1^ tends to decrease in intensity with increasing thermal activity, reflecting the formation and disruption of hydrogen bonds. This temperature-dependent behaviour is linked to changes in molecular configuration, and a weakening of the ∼3390 cm^−1^ band may indicate progressive dehydration or de­hydroxy­lation of the structure.

**Figure 6:**
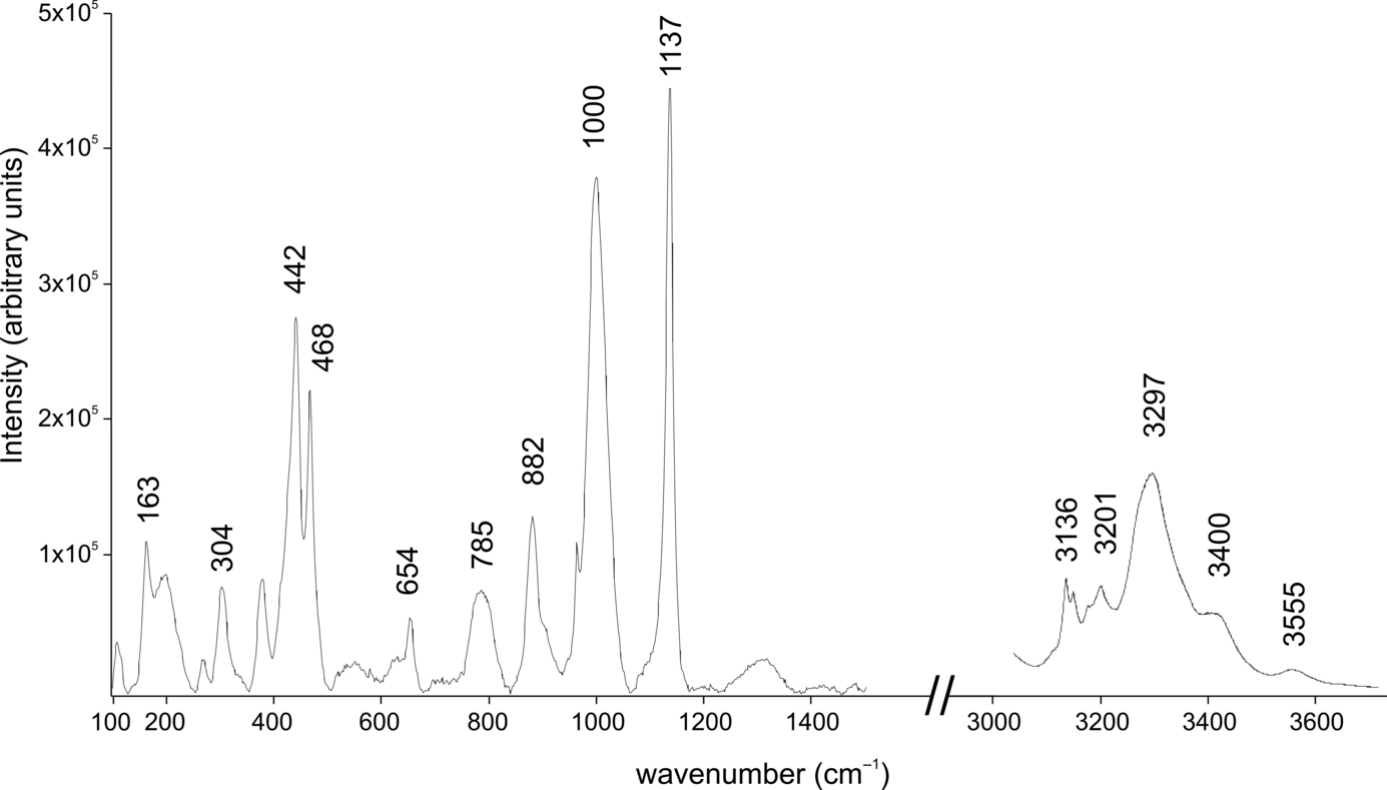
The Raman spectrum of synthetic *^C^*Na analogue of turkestanite.

Raman spectroscopy (Fig. 6 ▸) supports the presence of H_2_O in the Na-Tkt structure via the characteristic band at 3293 cm^−1^, typical for O—H stretching vibrations (Libowitzky, 1999 ▸). Assuming that oxygen from H_2_O occupies the *C* site, the resulting oxygen–oxygen distance is 2.728 (8) Å (Table 3 ▸), which corresponds to the formation of a moderately strong hydrogen bond. These values [3293 cm^−1^ and 2.728 (8) Å] ideally align with the correlation relating O—H stretching vibrations to O—H⋯O hydrogen-bond lengths in minerals (Libowitzky, 1999 ▸).

In the Raman spectrum, the strongest bands at 1137, 1000, 468 and 442 cm^−1^ are associated with stretching and bending vibrations in the [Si_8_O_20_]^8−^ cage. Eight corner-sharing tetrahedra forming this cage consist of a mixed system of bridging oxygen (BO) atoms and non-bridging oxygen (NBO) atoms. The bands at 1000 cm^−1^ and 1137 cm^−1^ in the Raman spectrum of Na-Tkt correspond to Si–O stretching vibrations involving BO and NBO, respectively. According to McMillan (1984 ▸), NBO-related bands typically appear at higher wavenumbers due to stronger and shorter Si—O bonds compared to those involving BO. In this study, the Si—O bond length for NBO is 1.571 (5) Å, while for BO it averages 1.620 (8) Å. The bands at 468 and 442 cm^−1^ are dominated by bending deformations of the double four-membered tetrahedral rings (McKeown, 2005 ▸; Bancroft *et al.*, 2023 ▸). The peaks at 785 cm^−1^ and 882 cm^−1^ are associated with the presence of alkali and alkaline metals (McMillan, 1984 ▸). The breathing motions of Si–BO–Si bridges are influenced by adjacent Na and Ca ions, leading to Na—O and Ca—O stretching vibrations (McKeown, 2005 ▸), while 654 cm^−1^ and 785 cm^−1^ may be attributed to symmetric Si–BO stretching (Bancroft *et al.*, 2023 ▸). Peaks up to 163 cm^−1^ are likely associated with lattice vibrations, while the peak at 304 cm^−1^ is probably related to *M*–O vibrations.

Based on SCXRD and refinement of the *C*-site occupancy, it might be inferred that there is no vacancy in Na-Tkt. The refined value of site scattering is 11.32 electrons per formula unit (epfu) (Table 4 ▸), which could correspond to a site population of 0.96 Na + 0.04 K. However, bond valence analysis (Brown, 2002 ▸; Brown & Altermatt, 1985 ▸; Gagné & Hawthorne, 2015 ▸) yields a sum of only 0.6 valence units (vu) for this site (Table 4 ▸). Such a low value suggests either a vacancy or, more probably that H_2_O occupies the *C* site, with the latter also consistent with the scattering value of 11.32 electrons pfu at this site (Table 3 ▸).

### 4.4. Structure of ^*C*^Na analogue of turkestanite/steacyite

Given the extremely low K content, the high Na content and possible presence of H_2_O in the phase resulting from experiment CF-15 (see below), it was important to determine its crystal structure and its relationships to Tkt, Scy and ekanite using SCXRD.

**Figure 7:**
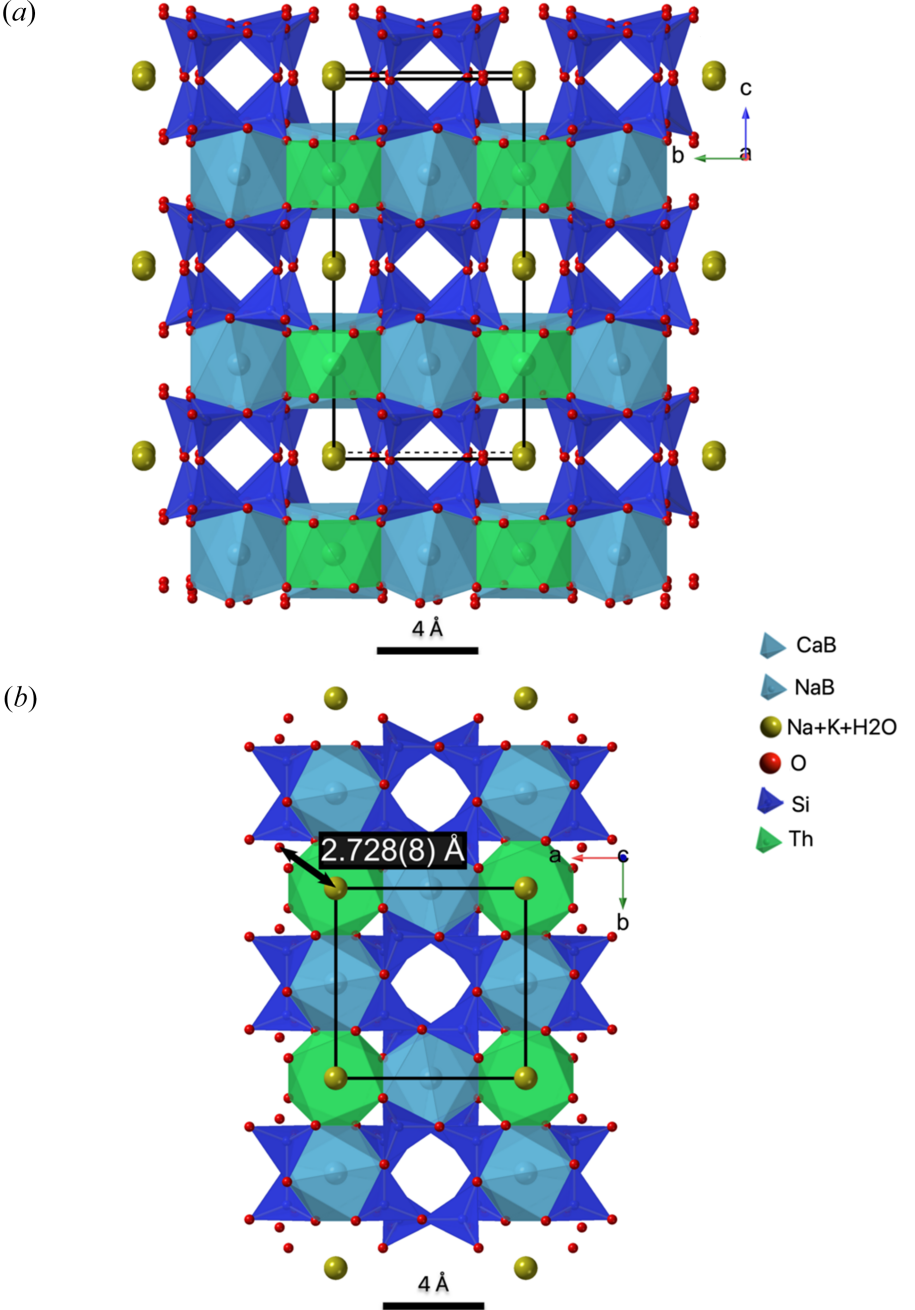
The crystal structure of the *^C^*Na analogue of turkestanite. The view along [100] (*a*); view along [001] (*b*). The *C* site is occupied mainly by Na together with K and H_2_O and is free of vacancy. The distance from the *C* site atoms to the closest neighbouring O atoms is shown, providing sufficient space for H_2_O molecules to form hydrogen bonds.

Na-Tkt is isostructural with turkestanite, steacyite and arapovite. In its crystal structure, SiO_4_ tetrahedra form an [Si_8_O_2__20_]^8−^ cage composed of two interconnected four-membered rings. Eight-coordinated *A* and *B* polyhedra share common edges to form a (001) sheet (Fig. 7 ▸). The sheets are connected through [Si_8_O_20_]^8−^ cages to form a framework. Atoms in the 12-coordinated *C* site fill large cages within the framework (Uvarova *et al.*, 2004 ▸). The absence of K in the structure and its replacement by Na results in a shortening of the *a* axis of the unit cell. However, unlike natural Scy and Tkt, the *C* site appears to be predominantly occupied by Na, with the remaining vacancy filled by H_2_O. The absence of vacancies caused an elongation of the unit cell *c* axis by approximately 0.2 Å. The crystal structures of Tkt and Scy have larger unit-cell volumes by ∼10 Å^3^ (1.4%). This is caused by the presence of K, which has a much larger ionic radius of 1.64 Å compared to Na (1.39 Å) despite the partial vacancy in their *C* site.

This study shows that, despite equal molar amounts of Na and Ca being introduced in experiment CF15, both *^C^*Na analogues of Scy and Tkt formed in comparable quantities (based on EPMA). In the *B* site, the Na:Ca occupancy ratio is close to 1:1, with a slight predominance of *^B^*Na over *^B^*Ca on average. When both the *C* and *B* sites are considered together, the average Na:Ca ratio approaches 2:1, indicating a general preference for Na incorporation into the structure during crystallization.

Structural analysis of Th-bearing Tkt and Scy cannot be easily obtained from natural samples due to the fact that natural Tkt and Scy tend to be somewhat metamict. This is shown by the very low residual electron densities (this study) compared to published studies for natural crystals of Tkt. The residual electron density peaks for Na-Tkt from this study are 1.09 e Å^−3^ (located 0.42 Å from *^A^*Th) and −1.37 e Å^−3^ (located 0.98 Å from *^A^*Th). In contrast, Kaneva *et al.* (2023 ▸) reported high residual peaks of 4.7 e Å^−3^ at ∼0.65 Å from the *^A^*Th position and ∼2.1 e Å^−3^ at ∼0.78 Å from the Si position for natural Tkt. Modelling these two residual peaks as O atoms led to physically unacceptable results. The residual maximum of 3.4 e Å^−3^, located near the *^A^*U position was noted by Uvarova *et al.* (2004 ▸) in the refinement of isostructural arapovite. Additionally, the presence of other phases within the Tkt crystal was noted (Kaneva *et al.*, 2023 ▸). The authors observed that the reflections of these phases might partly overlap with those of Tkt, resulting in a high *R*_int_ value of 17.8%. These difficulties highlight the limitations of the single-crystal method when applied to multi-phase samples.

## 5. Summary

The sequence of events in this study highlights the complementary nature of each analytical technique employed, *i.e.* EPMA, EBSD, Raman spectroscopy and SEM + FIB + SCXRD, regarding the characterization of the *^C^*Na analogue of turkestanite formed during the experimental alteration of chevkinite. The crystal separation procedure utilizing SEM + FIB eliminates the multiphase problem in crystal selection. Additionally, it allows for the analysis utilizing a carbon fibre of very small crystals, which are difficult or impossible to separate using an optical microscope. This has allowed for an in-depth analysis of the distribution of ions and H_2_O content in the synthetic *^C^*Na analogues of turkestanite and steacyite, which confirms the probable formation of *^C^*Na-turkestanite, ^A^Th*^B^*(CaNa)*^C^*Na(Si_8_O_20_), and *^C^*Na-steacyite, ^A^Th*^B^*(NaCa)*^C^*Na(Si_8_O_20_). Should either phase be found naturally it would constitute a new mineral. Here, Na occupies the crystallographic *C* site, which has not been recognized in the earlier studies for either turkestanite (Kaneva *et al.*, 2023 ▸; Kabalov *et al.*, 1998 ▸) or steacyite (Richard & Perrault, 1972 ▸) or isostructural arapovite (Uvarova *et al.*, 2004 ▸), as these phases contained insufficient Na content for this site to be considered. The vacancy, *^C^*□ can be filled by H_2_O during crystallization.

## Supplementary Material

Crystal structure: contains datablock(s) I. DOI: 10.1107/S2052520625004822/dk5136sup1.cif

Structure factors: contains datablock(s) I. DOI: 10.1107/S2052520625004822/dk5136Isup2.hkl

Supplementary Tables: raw analyses section and Fig. S1. DOI: 10.1107/S2052520625004822/dk5136sup3.xlsx

CCDC reference: 2454759

## Figures and Tables

**Table 1:** Chemical composition of synthetic *^C^*Na analogues of turkestanite (Na-Tkt) and steacyite (Na-Scy) from electron probe microanalyses Al, Pb and Mg have also been measured, all below detection (b.d.). * indicates total iron expressed as Fe^+^ in the calculations.

	Analysis number						
wt%	1	3	5	9	6	11	12
SiO_2_	56.08	55.35	55.49	56.36	54.75	56.15	54.76
TiO_2_					0.11	0.22	0.16
ZrO_2_					b.d.	0.19	0.41
ThO_2_	28.88	30.35	28.72	28.52	29.89	27.76	26.65
UO_2_	0.81	0.75	1.07	1.63	0.80	0.60	1.81
La_2_O_3_	0.11	b.d.	0.14	b.d.	0.11	0.28	b.d.
Ce_2_O_3_	0.31	0.20	0.55	0.23	0.20	0.77	0.40
Nd_2_O_3_	0.20	0.21	0.42	0.16	b.d.	0.45	0.23
CaO	5.75	5.01	6.17	6.03	5.53	5.64	4.56
MnO	0.34	0.37	0.54	0.57	0.29	1.52	2.50
FeO*	0.11	bd	0.14	b.d.	0.27	0.20	0.17
SrO	0.11	0.11	b.d.	b.d.	0.14	b.d.	0.08
Na_2_O	6.22	6.68	5.85	6.17	6.80	6.47	6.97
K_2_O	0.41	0.38	0.45	0.45	0.44	0.29	0.20
Total	99.33	99.41	99.54	100.12	99.33	100.54	98.90
							
	Formulae on the basis of 20 oxygens						
Si	8.10	8.07	8.05	8.10	8.01	8.04	8.05
Th	0.95	1.01	0.95	0.93	1.00	0.90	0.89
U	0.03	0.02	0.03	0.05	0.03	0.02	0.06
***^A^*Σ**	**0.97**	**1.03**	**0.98**	**0.99**	**1.02**	**0.92**	**0.95**
La	0.01	0.00	0.01	0.00	0.01	0.01	0.00
Ce	0.02	0.01	0.03	0.01	0.01	0.04	0.02
Nd	0.01	0.01	0.02	0.01	0.00	0.02	0.01
Ti					0.01	0.02	0.02
Zr					0.00	0.02	0.04
Ca	0.89	0.78	0.96	0.93	0.87	0.87	0.72
Mn	0.04	0.05	0.07	0.07	0.04	0.18	0.31
Fe^2+^	0.01	0.00	0.02	0.00	0.03	0.02	0.02
Sr	0.01	0.01	0.00	0.00	0.01	0.00	0.01
Na	1.01	1.14	0.90	0.98	1.02	0.82	0.87
***^B^*Σ**	**2.00**	**2.00**	**2.00**	**2.00**	**2.00**	**2.00**	**2.00**
Na	0.73	0.75	0.75	0.74	0.91	0.97	1.11
K	0.08	0.07	0.08	0.08	0.08	0.05	0.04
***^C^*Σ**	**0.80**	**0.82**	**0.83**	**0.82**	**0.99**	**1.02**	**1.15**
**Σ cations**	**11.87**	**11.92**	**11.87**	**11.91**	**12.03**	**11.99**	**12.13**
**Phase**	**Na-Scy**	**Na-Scy**	**Na-Tkt**	**Na-Tkt**	**Na-Scy**	**Na-Tkt**	**Na-Tkt**

**Table 2:** Experimental details

Crystal data	
Chemical formula	Ca_1.105_K_0.038_Na_1.858_O_20_Si_8_Th_0.979_
*M*_r_	860.73
Crystal system, space group	Tetragonal, *P*4/*mcc*
Temperature (K)	297
*a*, *c* (Å)	7.4757 (2), 14.9658 (7)
*V* (Å^3^)	836.38 (6)
*Z*	2
Radiation type	Mo *K*α
μ (mm^−1^)	9.82
Crystal size (mm)	0.01 × 0.01 × 0.005

Data collection	
Diffractometer	SuperNova, Single source at offset/far, Eos
Absorption correction	Multi-scan (*CrysAlis PRO*). Empirical absorption correction using spherical harmonics, implemented in SCALE3 ABSPACK scaling algorithm.
*T*_min_, *T*_max_	0.845, 1.000
No. of measured, independent and observed [*I* > 2σ(*I*)] reflections	2690, 519, 411
*R*_int_	0.084
(sin θ/λ)_max_ (Å^−1^)	0.663

Refinement	
*R*[*F*^2^ > 2σ(*F*^2^)], *wR*(*F*^2^), *S*	0.035, 0.067, 1.06
Final *R* indexes [*I* > = 2σ(*I*)]	*R*_1_ = 0.0354, *wR*_2_ = 0.0612
Final *R* indexes (all data)	*R*_1_ = 0.0546, *wR*_2_ = 0.0656
No. of reflections	519
No. of parameters	44
Δρ_max_, Δρ_min_ (e Å^−3^)	1.09, −1.37

Computer programs:*CrysAlis PRO* (Rigaku OD, 2023 ▸), *SHELXT* (Sheldrick, 2015*a* ▸), *SHELXL2019/2* (Sheldrick, 2015*b* ▸), *OLEX2* (Dolomanov *et al.*, 2009 ▸).

**Table 3:** Refined site-scattering values as electrons pfu and assigned site populations for synthetic *^C^*Na analogue of turkestanite

Site	Refined site scattering	Site population	Calculated site scattering
*A*	87.3	0.94 Th + 0.03 U	87.36
*B*	31.9	0.96 Na + 0.90 Ca + 0.02 Ce + 0.01 Nd + 0.1 Mn	31.31
*C*	11.32	0.84 Na + 0.07 K + 0.1 O	11.37

**Table 4:** Bond-valence calculations for synthetic *^C^*Na analogue of turkestanite (valence units) Bond valence parameters were taken from Gagné & Hawthorne (2015 ▸).

	O1	O2	O3	BV sum
*^A^*Th			0.5135^8→↓^	4.0217
^B^(Ca_0.55_, Na_0.45_)		0.1220^4→↓^	0.2446^4→↓^	1.4663
*^C^*(Na_0.96_K_0.04_)	0.0893^4→↓^		0.0299^8→↓^	0.5966
Si	1.0208^→^^2↓^	1.0260^→↓^	1.1460^→↓^	4.1724
		0.9796^→↓^		
BV sum	2.1308	2.1277	1.9232	

## Data Availability

Raw data and detailed experimental procedures can be accessed upon reasonable request from the corresponding author.
